# Targeted PPARδ activation reprograms microglial immunometabolism and improves insulin sensitivity in HFD-fed rats

**DOI:** 10.1016/j.jlr.2026.100978

**Published:** 2026-01-08

**Authors:** Han Jiao, Fernando Cázarez-Márquez, Valentina Sophia Rumanova, Yalin Wang, Andries Kalsbeek, Gertjan Kramer, Shanshan Guo, Chun-Xia Yi

**Affiliations:** 1 Department of Endocrinology and Metabolism, Amsterdam University Medical Center, University of Amsterdam, Amsterdam, The Netherlands; 2 Amsterdam Gastroenterology Endocrinology and Metabolism, Amsterdam, The Netherlands; 3 Department of Clinical Chemistry, Laboratory of Endocrinology, Amsterdam University Medical Centers, Amsterdam, The Netherlands; 4 Netherlands Institute for Neuroscience, Amsterdam, The Netherlands; 5 Department of Arctic and Marine Biology, UiT The Arctic University of Norway, Tromsø, Norway; 6 Key Laboratory of Quantitative Synthetic Biology, Shenzhen Institute of Synthetic Biology, Shenzhen Institutes of Advanced Technology, Chinese Academy of Sciences, Shenzhen, China; 7 Department of Mass Spectrometry of Biomolecules, Swammerdam Institute for Life Sciences, University of Amsterdam, Amsterdam, The Netherlands; 8 Faculty of Synthetic Biology, Shenzhen University of Advanced Technology, Shenzhen, China

**Keywords:** Microglia, immunometabolism, PPARδ, insulin sensitivity, nanoparticles, obesity

## Abstract

Microglia lipid metabolism plays a crucial role in maintaining immune function and supporting neuronal health. Previous studies have shown that a high-fat diet (HFD) promotes lipid accumulation in microglia, while disruption of lipid uptake and utilization impair neuroimmune competency and accelerate obesity in response to a HFD, highlighting the importance of lipid processing under obesogenic conditions. However, whether enhancing microglial lipid metabolism can restore their immune function and mitigate obesity-associated hypothalamic dysfunction remains unclear. In this study, we investigated whether activation of peroxisome proliferator-activated receptor delta (PPARδ), a key regulator of lipid metabolism, could counteract obesity-related metabolic disturbances. Using thermal proteome profiling, we identified GW0742 as the most potent PPARδ agonist among those tested. Treatment of microglial cells *in vitro* with GW0742 enhanced phagocytosis, reduced inflammation, and improved microglial metabolic flexibility. To assess therapeutic potential *in vivo*, we selectively delivering GW0742 to mediobasal hypothalamic microglia in HFD-fed rats using polymeric nanoparticles (NPs-GW0742). This targeted intervention reprogrammed microglial activity and improved insulin sensitivity without affecting body weight or food intake, suggesting a direct central metabolic benefit. Our findings highlight the therapeutic potential of targeting microglial lipid metabolism to improve metabolic health in obesity.

## Graphical Abstract

**Figure:**
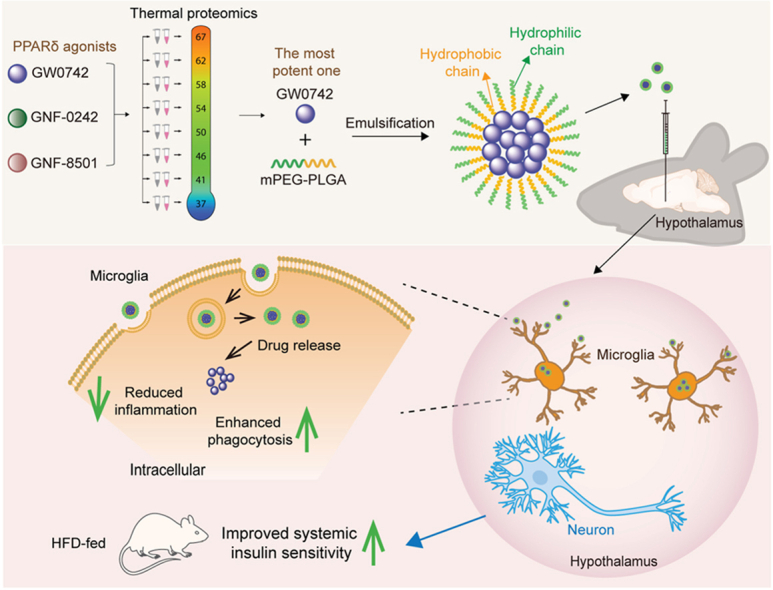

Microglia are the primary innate immune cells of central nervous system (CNS), playing a crucial role in maintaining a healthy microenvironment and supporting neuronal health (1, 2). Dysfunction of microglia in the hypothalamus has been closely linked to obesity, particularly in the context of a high-fat diet (HFD) (3, 4). Emerging evidence suggests that intracellular metabolism is tightly coupled to immune cell function (5, 6). For example, HFDs promote lipid accumulation in microglia (7), and loss of lipoprotein lipase, a key enzyme involved in lipid uptake and utilization, impairs microglial immune responsiveness and accelerates obesity under HFD conditions (8, 9). These findings suggest that, rather than suppressing microglial lipid metabolism, strategies aimed at enhancing it may offer a promising therapeutic avenue to reprogram microglial activity and support their role in combating obesity and its associated metabolic disorders.

The peroxisome proliferator-activated receptor (PPAR) family comprises three isoforms, PPARα, PPARγ and PPARδ, each of them playing a key role in regulating lipid metabolism and maintaining energy homeostasis (10, 11). In the CNS, the distribution and expression of PPARs are both region- and cell-type specific. Regionally, PPARα is predominantly localized in the hippocampus, cerebellum, olfactory bulb, and retina, whereas PPARγ has a more restricted distribution (12, 13). Notably, PPARδ is highly expressed in the mediobasal hypothalamus (MBH), a critical brain region involved in eating behaviour and energy regulation (13, 14). At the cellular level, all three PPAR isoforms are expressed in neurons, while astrocytes primarily express PPARα and PPARγ (15). Importantly, microglia exhibit the highest levels of PPARδ expression among brain cell types (16, 17). Previous study shows that PPARδ-deficient mice on a HFD display impaired thermogenesis and an increased susceptibility to obesity (18). Furthermore, brain-wide deletion of PPARδ in microglia has been associated with reduced cell proliferation and elevated production of proinflammatory cytokines following lipopolysaccharide (LPS) stimulation in an experimental autoimmune encephalomyelitis mouse model (19). Despite these findings, the specific consequences of microglia-targeted PPARδ activation in the MBH, particularly its influence on immune and metabolic functions are still poorly understood.

Besides this, although GW0742 is one of the most widely used and well-characterized PPARδ agonists (20), several newly developed agonists - such as GNF-0242 and GNF-8501 - have recently emerged (21). However, their relative efficacy compared with GW0742 and their effects have not been systematically evaluated in microglia. To address this gap, we first employed thermal proteome profiling (TPP) (21, 22) to assess the target engagement and binding properties of GW0742 alongside the two novel PPARδ agonists, GNF-0242 and GNF-8501. We then performed *in vitro* immunometabolic assays to compare their effects on key microglial functions. Based on these analyses, the most potent compound, GW0742, was selected for *in vivo* testing by selectively delivered to microglia in the MBH of HFD-fed male Wistar rats. This allowed us to assess its impact on food intake, body weight, and systemic metabolism. To achieve microglia-specific delivery of GW0742, we developed a nanoparticle (NP)-based system, building on our previous work using NPs to deliver siRNA into hypothalamic microglia for genetic manipulation (23). This strategy enabled selective activation of PPARδ in microglia in the MBH, providing a unique opportunity to investigate its role in regulating metabolic and immune processes. By integrating TPP, *in vitro* functional assays, and NP-mediated targeted delivery, our study underscores the therapeutic potential of microglial PPARδ activation in the MBH in alleviating obesity-related metabolic dysfunction.

## Materials and Methods

### Cell viability assay

According to the manufacturer's instructions, cell viability was assessed using the Cell Counting Kit-8 (CCK-8, MCE, Cat. No.: HY-K0301). Specifically, cells were seeded into 96-well plates at a density of 5,000 cells per well, treated with different dose of PPARδ agonists, and then incubated with 10 μl of CCK-8 solution per well. After 1 h of incubation, the optical density at 450 nm was measured using a microplate reader.

Previous studies using tool cell lines have shown that the EC_50_ values of GNF-0242 (AKos GmbH, Z107275398), GNF-8501 (AKos GmbH, Z104661894), and GW0742 (Sigma-Aldrich, G3295-5MG) are 0.004 μM, 0.006 μM, and 0.001 μM (21, 24), respectively. However, EC_50_ values can vary substantially depending on cellular context, receptor abundance, and co-factor availability (25). Consistent with this, studies in glial cells or animals have frequently employed higher concentrations to achieve functional PPARδ activation (26, 27). For instance, GW0742 has been used at 1 μM in astrocytes (26), suggesting that effective concentrations in glial populations may exceed those reported in tool cell lines.

Therefore, prior to subsequent experiments, we determined the appropriate effective concentration in microglial cells by performing a dose-dependent cell viability assay using the cell counting kit-8 (CCK8), testing a range of 0.0001 μM, 0.001 μM, 0.01 μM, 0.1 μM, 1 μM, and 10 μM (Supplemental Fig. S1A–C).

### Cell culture and treatment

BV2 microglial cells were cultured in DMEM with 10% FBS and 1% PSN at 37°C in 5% CO_2_. Upon reaching 80–90% confluency, cells were treated with 1 μM PPARδ agonists, including GNF-0242, GNF-8501, and GW0742 or 0.05% DMSO as control. For LPSstimulation, treatments were applied in serum-free MEM with or without 100 ng/ml LPS for 6 h and 24 h. After treatment, cells were washed with PBS and collected for further analysis. In brief, total RNA from BV2 cells was extracted, and gene expression was performed by RT-PCRs. Primer sequences can be found in Supplemental Table S1. And for cytokine concentration, culture media were collected at 24 h, centrifuged at 3,000 g for 15 min, and the supernatants were dispatched to Servicebio for quantification of TNF-α and IL-6 via ELISA.

### Sample preparation for thermal proteome profiling (TPP)

The samples were prepared as presented previously with minor modifications (28). In brief, the cells were incubated for 5 h at 37°C, 5% CO_2_ in an incubator. After treatment, the cells were detached with cell scraping and the cell suspension was collected in 50 ml tubes. The cells were centrifuged (300 g, 3 min) to pellet the cells. And the cell pellets were resuspended in PBS containing protease inhibitors and divided into 8 tubes. The intact cells were then heated for 3 min at designated temperatures (37–67°C), incubated at room temperature for 3 min, and snap-frozen in liquid nitrogen. Thermally treated pellets were lysed by three freeze-thaw cycles in 100 mM triethylammonium bicarbonate (TEAB) buffer, followed by centrifugation to remove aggregates. Protein concentration was measured via BCA assay, and 20 μg of protein (based on the 37°C sample) was reduced, alkylated, and cleaned via Single-Pot Solid-Phase-enhanced Sample Preparation as described previously (29). Samples were digested overnight with trypsin (1:20 w/w), purified using OASIS HLB plates, and vacuum dried. Peptides were reconstituted in 1 M TEAB and labeled with *iTRAQ* (8-plex kit, Thermo Fisher Scientific). Raw data of TPP can be found in Supplemental Tables S2–S5.

### Fractionation chromatography and mass spectrometry for thermal proteome profiling

Pooled *iTRAQ*-labeled peptides were lyophilized, reconstituted in solvent A (10 mM ammonium formate, 20% acetonitrile in ULC-MS water), and subjected to strong cation exchange chromatography using a PolySULFOETHYL Aspartamide SCX column (2.1 mm × 10 cm, PolyLC) on an Agilent 1100 HPLC system with a micro-fraction collector. Peptides were eluted with a linear gradient from 0% to 100% solvent B (500 mM ammonium formate, 20% acetonitrile) and collected in 1-min intervals from 5 to 40 min. Fractions were combined into eight pools and lyophilized for mass spectrometry. Dried samples were reconstituted in 0.1% formic acid (ULC-MS grade, Biosolve), and 200 ng of peptides were injected onto a C18 column (75 μm × 250 mm, 1.6 μm, Ionopticks) maintained at 50°C. Separation was performed using a NanoRSLC Ultimate 3000 UHPLC (Thermo Scientific) with a multi-step gradient (3–99% solvent B: 0.1% formic acid in acetonitrile) over 60 min at 400 nl/min. Peptides were ionized using a captive-spray source and analyzed on a timsTOF Pro (Bruker) in PASEF mode (m/z 100–1700; TIMS range: 0.6–1.6 V s/cm^2^), collecting 10 MS/MS scans per 1.16-s cycle. Instrument settings followed Ogata *et al*. (2021) for optimal isobaric tag detection (30).

### Glucose uptake and phagocytosis assays in BV2 cells

2-NBDG glucose uptake assay was using the method described before (31). In Brief, BV2 cells were treated with 1 μM PPARδ agonists or DMSO for 6 h, then incubated with 200 μg/ml 2-NBDG (Abcam, ab235976) for 1 h for glucose uptake analysis, after that, the plate was washed twice with warm PBS before sending to a fluorescence plate reader (Ex/Em: 480/530 nm). For phagocytosis, BV2 cells were exposed to 0.01% fluorescent microspheres (Sigma-Aldrich, L1030-1 ml) pre-coated in 10% FBS for 1 h at 37°C. A negative control was included by incubating cells with microspheres for 1 h at 4°C, as described previously (32, 33, 34). Following incubation, cells were washed thoroughly with cold PBS to remove non-internalized beads and fixed with 4% paraformaldehyde. Phagocytic activity was assessed using confocal microscopy.

### Immunocytochemistry and BODIPY staining

For immunocytochemistry of BV2 microglia in coverslips, in brief, coverslips were pre-coated with poly-L-lysine (0.5 mg/ml, Sigma-Aldrich, P9155) according to the manufacturer’s instructions. Fixation was carried out for 15 min at room temperature after which the coverslips were washed thoroughly with 3× PBS. Immunostaining was performed overnight at 4°C using primary antibodies: Guinea pig anti-IBA1 (synaptic system, HS234808, 1:500), Rabbit anti-TNF-α (Abcam, ab205587, 1:400). Sections were then incubated for 1 h at room temperature with a secondary anti-guinea pig conjugated with biotin antibody (Vector, cat. BA7000, 1:400). After that, sections were incubated for 2 h at room temperature with: Donkey anti-rabbit Alexa 594 (Invitrogen, A-21207, 1:400), Streptavidin Alexa Fluor 594 conjugate (Invitrogen, S32356, 1:800) and Streptadivin Alexa Fluor 647 (Invitrogen, S32357, 1:800). Sections were then washed once in TBS and incubated in TBS with BODIPY™ 493/503 (1:1,000 from 1 mg/ml stock solution in DMSO; ThermoFisher) to stain lipid droplets and with DAPI (Thermo Fisher, cat. 62247, 1:2,000) for nuclear counterstaining for 20 min at room temperature. Sections were mounted on microscope slides and embedded with Vectashield (H-1000, Vector Laboratories).

### Seahorse measurement

BV2 cells (5,000 cells per well) were treated with 1 μM GW0742 or DMSO for 24 h, then assessed for oxygen consumption rate (OCR) using the Seahorse XF Cell Mito Stress Test. Basal respiration and mitochondrial function were measured following injections of oligomycin (1.5 μM), FCCP (0.5 μM), and rotenone (1.25 μM)/antimycin A (2.5 μM). For glycolysis, extracellular acidification rate (ECAR) was measured in glucose/pyruvate-free media using the XF Glycolysis Test with glucose (10 mM), oligomycin (1 μM), and 2-deoxyglucose (50 mM). Data were normalized to protein content quantified by the Bradford assay.

### Synthesis and characterization of nanoparticles

Biocompatible and biodegradable methoxy poly(ethylene glycol)-poly(lactic-co-glycolic acid) (mPEG-PLGA) nanoparticles were synthesized to deliver GW0742. Using an emulsion/solvent evaporation method, copolymer and GW0742 were dissolved in dichloromethane, emulsified in polyvinyl alcohol, and processed by solvent evaporation and centrifugation. Nanoparticles were characterized by size, polydispersity index, and zeta potential (Zeta Sizer Nano ZS), and morphology was assessed by transmission electron microscopy. GW0742 encapsulation efficiency was determined via HPLC after nanoparticle disruption and calculated as a percentage of drug encapsulated relative to the initial amount.

### *In vitro* stability, encapsulation efficiency, and drug release

To assess stability, 50 mg of blank NPs were suspended in PBS at pH 7.4 and pH 4.4 and incubated at 37°C with constant stirring at 110 rpm. The particle size was measured on Days 0, 1, 2, and 3 using dynamic light scattering. To evaluate encapsulation efficiency (EE), NPs-GW0742 were dissolved in dichloromethane (DCM) to disrupt the polymeric shell and release the encapsulated drug. DCM was removed via rotary evaporation, and the remaining GW0742 was dissolved in acetonitrile. Drug concentration was quantified using high-performance liquid chromatography (HPLC; SPD-20A/20AV Series, equipped with a SIL-20A/20AC detector and a Thermo Scientific C18 reversed-phase column, Part No. 25005–154630). The detection wavelength was set at 226 nm. A standard curve was generated using serial dilutions of GW0742. Encapsulation efficiency was calculated using the formula: EE (%) = (A/B) × 100, where A is the amount of GW0742 encapsulated in the nanoparticles and B is the total amount of GW0742 added during synthesis.

### Animals

Male Wistar rats (Charles River, Germany), aged 8–10 weeks (body weight: 150–200 g), were housed under a 12-h light/12-h dark cycle (lights on at 7:00 a.m.) at a controlled temperature of 22 ± 2°C, with ad libitum access to food and water. All rats were fed a HFD (Research Diet, D12331, 58% kcal fat, 25.5% kcal carbohydrate as sucrose) for 10 weeks before undergoing surgery. All animal procedures were conducted in accordance with the guidelines on animal experimentation of the Netherlands Institute for Neuroscience (NIN) and were approved by the Animal Ethics Committee of the Royal Dutch Academy of Arts and Sciences (KNAW).

### Surgery

Rats were anesthetized with ketamine (80 mg/kg) and xylazine (8 mg/kg) for vessel cannulation and brain infusion probe implantation, with anesthesia carefully monitored. Using a Kopf stereotaxic apparatus, bilateral brain infusion probes were precisely placed into the mediobasal hypothalamus (coordinates: AP −2.8 mm, ML ±2.0 mm, angle 8°, DV −10 mm). For targeting visualization, 1 μl of Rhodamine B (RhoB)-labeled nanoparticles was injected per side via a Hamilton syringe. For long-term nanoparticle delivery and blood sampling, catheters and bilateral guide cannulas were implanted. After recovery, rats received daily bilateral injections of control or GW0742-loaded nanoparticles (1 μM) for 12 days at 4 p.m. Injectors remained in place for 3 min to avoid backflow.

### Endogenous glucose production and insulin tolerance test

Endogenous glucose production (EGP) and insulin tolerance tests (ITT) were performed as described previously (35). After fasting, rats received NPs infusion in the hypothalamus. [6,6-^2^H_2_]-D-glucose was infused for 90 min to reach steady state, enabling EGP calculation from blood samples. Insulin (0.25 IU/kg) was then injected for ITT, with glucose measured at multiple time points. Blood was collected for glucose and hormone assays, and animals were refed after testing. Plasma [6,6-^2^H_2_]-D-glucose enrichment was measured by GC-MS and EGP was calculated using the methods of Steele (36). Insulin (Millipore) levels were determined by radio-immunoassays according to the manufacturer’s instructions.

### Indirect calorimetry

One day after the EGP and ITT, rats were single-housed in metabolic cages (TSE system) for 4 days. Food and water intake, locomotor activity, respiratory exchange ratio, and energy expenditure were continuously monitored and recorded.

### Plasma triglycerides and cholesterol measurement

Plasma triglycerides and cholesterol levels were measured by enzymatic colorimetric tests (Triglycerides liquicolor^mono^, No. 10724 and Cholesterol liquicolor, No. 10028, HUMAN Diagnostics) according to the manufacturer’s instructions.

### Brain collection and immunostaining

Following four days of metabolic monitoring, rats were euthanized and perfused with saline followed by 4% paraformaldehyde. Brains were extracted, post-fixed, and sectioned for immunofluorescence or immunohistochemistry. For immunofluorescence, sections were stained for anti-Iba1 (Synaptic Systems, No. 234003; 1:400), anti-GFAP (DAKO, Z0334; 1:400), and anti-orexin (Abcam, Ab6214; 1:1,000) to assess NPs-RhoB localisation. Sections were mounted with antifade media and imaged using Leica TCS SP8 confocal microscopy. For microglial evaluation, immunohistochemical staining was performed on 2-3 sections per brain using the Iba1 antibody (Synaptic Systems, No. 234003; 1:2,000). Sections were dehydrated, cleared, and cover-slipped before imaging with a Zeiss Axioplan microscope. Microglial cell count, soma size, and coverage in the arcuate nucleus were quantified using Fiji software. Results were averaged per rat for subsequent analysis.

### Statistical analysis

Data were presented as means ± SEM, unless otherwise stated. Group differences were assessed using two-tailed Student’s *t* test or one-way/two-way ANOVA followed by appropriate post hoc tests. Four animals were excluded from the EGP analysis due to blood vessel clotting. One animal per group was excluded from ITT analysis due to adverse reactions following insulin injection. Statistical significance was defined as *P* < 0.05 (two-sided). Analyses were performed using IBM SPSS Statistics v22 and GraphPad Prism.

## Results

### Thermal proteome profiling of GNF-0242, GNF-8501, and GW0742

Based on the results, GW0742 and GNF-0242 showed no detectable toxicity in microglia, whereas GNF-8501 exhibited only a modest reduction in viability (Supplemental Fig. S1A–C). Considering future *in vivo* applications and bioavailability, we selected a 1 μM as reported in other glia studies (27). Thus, we proceeded with 1 μM for all subsequent assays to ensure sufficient receptor engagement while avoiding cytotoxicity.

**Fig. 1:**
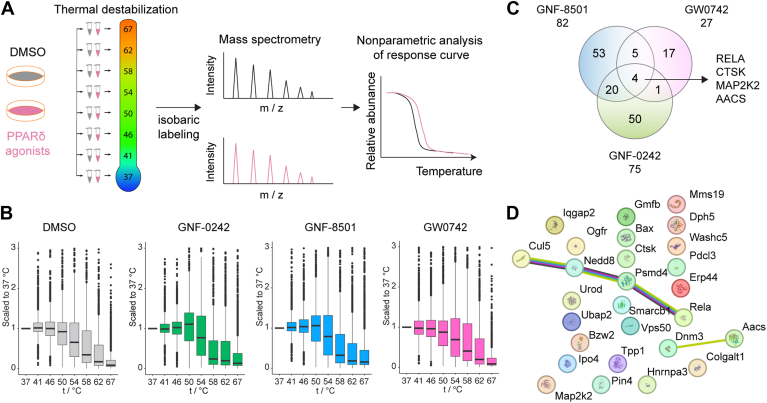
Thermal Proteome Profiling of PPARδ agonists treated microglial cells. A: Overview of the experimental setup. PPARδ agonists and vehicle (DMSO) treatment and 8-temperature destabilization were performed. n = 6. B: Distribution of TPP data after scaling each protein to the abundance signal in the lowest temperature condition (37°C). The solid line in the box plots represents the median, box limits show the interquartile range (IQR) and its whiskers 1.5 × IQR. Outliers are represented as block dots. C: Venn diagram representing the number of proteins with shifted temperatures, compared to control treatment for each of the three tested agonists - GNF-0242, GNF-8501, or GW0742. D: Protein networks with increased temperature induced by GW0742.

Afterward, we applied thermal proteome profiling (TPP) to investigate proteomic changes induced by the PPARδ agonists. TPP is a powerful method for evaluating protein thermal stability and abundance, enabling the unbiased identification of direct and indirect drug targets (37, 38). Unlike traditional proteomic approaches, TPP offers high throughput, sensitivity, precision, and comprehensive insights into complex biological systems (39). The key principle of TPP is that ligand binding increases the thermal stability of target proteins relative to their unbound state (40). We applied TPP to evaluate proteomic changes in microglia treated with the PPARδ agonists GNF-0242, GNF-8501, and GW0742 (Fig. 1). Proteins showing altered thermal stability compared to the vehicle control were identified as potential off-targets, reflecting reduced ligand specificity (Fig. 1A). Our analysis identified over 2,000 proteins with significant changes in thermal stability across the three PPARδ agonists (Fig. 1B). Specifically, GNF-0242, GNF-8501, and GW0742 induced significant melting point shifts in 75, 82, and 27 proteins, respectively (Fig. 1C). These results suggest that GNF-0242 and GNF-8501 interact with a wider range of proteins than GW0742, indicating a higher potential for off-target effects. Four proteins with altered melting points were shared by all three agonists: RELA, CTSK, and MAP2K2 showed increased melting temperatures, while AACS exhibited a decrease (Fig. 1C). For GW0742 specifically, 27 proteins displayed increased thermal stability. Functional enrichment analysis revealed that four of these proteins - CLU5, NEDD8, PSMD4, and RELA - are involved in pathways related to cellular stress response and protein degradation (Fig. 1D). These findings underscore the potential pathways modulated by GW0742 and indicate that GW0742 is a more selective PPARδ agonist compared to GNF-0242 and GNF-8501.

### GW0742 induces phagocytic and anti-inflammatory effects in microglia among the three PPARδ agonists

**Fig. 2:**
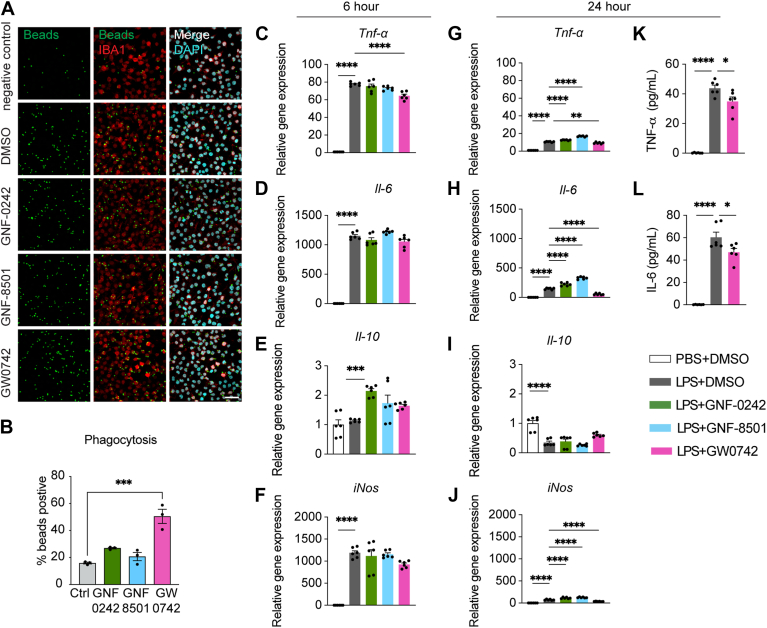
The effects of PPARδ agonists on microglial phagocytosis and immune response. A, B: The representative figures (A) and phagocytosis quantification (B) of microglia cells treated with DMSO or PPARδ agonists. Scale bar, 100 μm. Negative control group was incubated in 4°C for 1 h. C–F: Relative mRNA levels of cytokines (*Tnf-α*, *Il-6*, *Il-10*) and oxidative stress-related gene *iNos* in BV2 microglia treated with PPARδ agonists or DMSO together with 100 ng/ml LPS or PBS treatment for 6 h. G–J: Relative mRNA levels of genes in BV2 microglia treated with PPARδ agonists or DMSO together with 100 ng/ml LPS or PBS treatment for 24 h. K, L: The concentrations of TNF-α and IL-6 in the culture medium 24 h after treatment. Data are presented as means ± SEM and statistical significance was determined using Unpaired *t* test. ∗, *P* < 0.05; ∗∗, *P* < 0.01; ∗∗∗, *P* < 0.001; ∗∗∗∗, *P* < 0.0001.

Given the crucial role of microglial phagocytosis in neuroprotection, we assessed the effects of the three PPARδ agonists on microglial phagocytic activity. Our analysis revealed that only GW0742 significantly enhanced microglial phagocytic function compared to the DMSO control (Fig. 2A, B). To further evaluate how PPARδ agonists influence microglial immune responses, BV2 cells were treated with PPARδ agonists and subsequently challenged with LPS (100 ng/ml) for either a short (6 h) or a prolonged duration (24 h) (Fig. 2C–J). As expected, LPS exposure significantly increased the expression of pro-inflammatory cytokines tumor necrosis factor alpha (*Tnf-α*) (Fig. 2C, G) and interleukin-6 (*Il-6*) (Fig. 2D, H), as well as the oxidative stress marker inducible nitric oxide synthase (*iNos/Nos2*) (Fig. 2F, J) at both time points, while reducing the expression of the anti-inflammatory cytokine interleukin-10 (*Il-10*) exclusively on longer period (Fig. 2J). Among the three PPARδ agonists tested, only GW0742 consistently attenuated LPS-induced upregulation of *Tnf-α* at both 6 h and 24 h (Fig. 2C, G), suggesting its stronger anti-inflammatory efficacy. To further confirm these transcriptional changes, we measured TNF-α and IL-6 protein levels in the culture media after 24 h of LPS stimulation (Fig. 2K, L). Consistent with the mRNA results, GW0742 markedly reduced the LPS-induced secretion of both cytokines (Fig. 2K, L). Taken together, these results demonstrate that GW0742 is more effective than GNF-0242 and GNF-8501 in enhancing microglial phagocytosis and modulating microglial immune responses, underscoring its better potency as a PPARδ agonist.

### GW0742 modulates microglial energetic metabolism

**Fig. 3:**
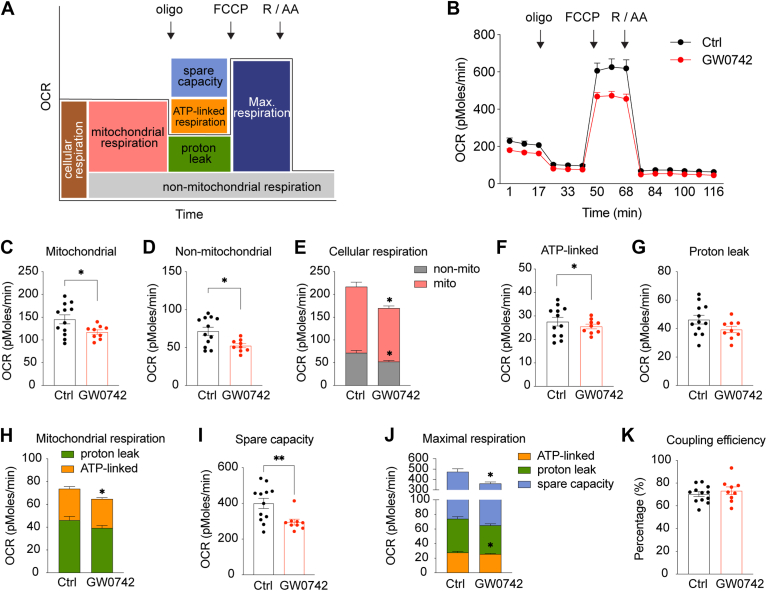
GW0742 reduces microglial ATP-linked respiration without changing coupling efficiency. A: Scheme defining the oxygen-consuming rate (OCR) processes of cells. Oligomycin (oligo); carbonyl cyanide-4 (trifluoromethoxy) phenylhydrazone (FCCP); rotenone (R) and antimycin A (AA). B: OCR over time in microglial cells treated with GW0742 or vehicle (Ctrl, DMSO). C–E: Cellular respiration was dissected into mitochondrial and nonmitochondrial respiration using the electron transfer chain inhibitors, rotenone and antimycin A. F–H: Mitochondrial respiration was further dissected into ATP-linked respiration and proton leak using the ATP-synthase inhibitor, oligomycin. I, J: Maximal respiration of cells after addition of the chemical uncoupler, FCCP, and after subtracting nonmitochondrial respiration rates. The portion of spare respiratory capacity was determined by subtracting basal respiration from maximal respiration rates. K: Mitochondrial coupling efficiencies were calculated as the fraction of mitochondrial oxygen consumption that is sensitive to oligomycin, reflecting the fraction used to drive ATP synthesis. Data are presented as means ± SEM and statistical significance was determined using Unpaired *t* test. ∗, *P* < 0.05; ∗∗, *P* < 0.01.

**Fig. 4:**
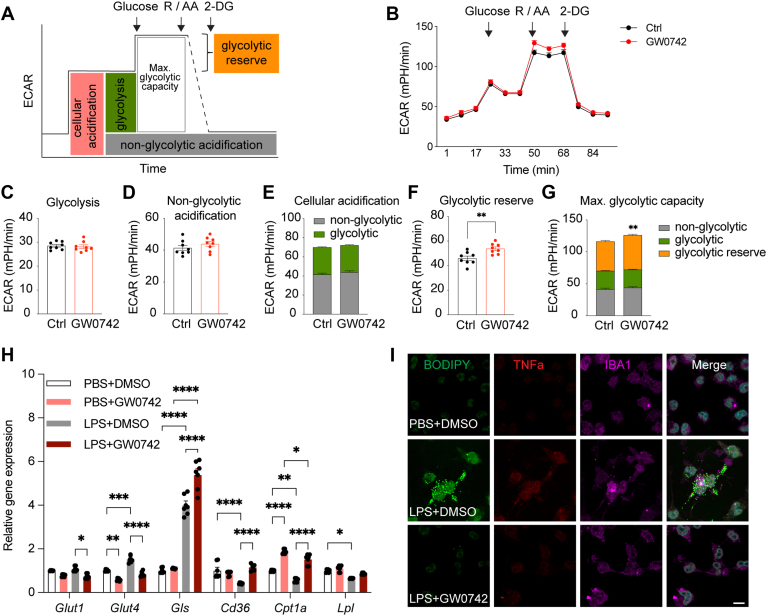
GW0742 alters microglial metabolic flexibility. A: Scheme defining the cellular extracellular acidification rate (ECAR) process. Rotenone (R); antimycin A (AA); 2-Deoxy-D-glucose (2-DG). B: The ECAR over time in BV2 microglial cells treated with GW0742 or vehicle (Ctrl, DMSO). C–E: Cellular acidification was dissected into glycolysis and non-glycolytic acidification. F, G: Glycolytic reserve and maximal glycolytic capacity. H: The expression of metabolism genes of Ctrl and GW0742 with or without LPS stimulation. I: The staining of BODIPY and TNF-α in treated microglial cells. Scale bar: 30 μm. Data are presented as means ± SEM and statistical significance was determined using Unpaired *t* test. ∗, *P* < 0.05; ∗∗, *P* < 0.01; ∗∗∗, *P* < 0.001; ∗∗∗∗, *P* < 0.0001.

Microglial immune function is closely linked to their metabolic state, referring to an interaction known as immunometabolism, which is essential for regulating inflammatory responses, maintaining neuronal homeostasis, and supporting overall brain function (5). To investigate the effects of GW0742 on microglial metabolic activity, microglia were treated with 1 μM GW0742 or DMSO (vehicle control) for 24 h, followed by assessments of oxygen consumption rate (OCR) and extracellular acidification rate (ECAR) using a Seahorse XFe96 analyzer to measure mitochondrial respiration and glycolysis, respectively. The traces for OCR and ECAR and the analyzed metabolic parameters are shown in Fig. 3A and 4A, with the corresponding time-lapse data presented in Fig. 3B and 4B. GW0742 treatment significantly reduced mitochondrial respiration (Fig. 3C–E), primarily due to a decrease in ATP-linked respiration, while the proton leak remained unchanged (Fig. 3F–H). Furthermore, GW0742 reduced maximal substrate oxidation capacity (Fig. 3I, J), while coupling efficiency showed no change (Fig. 3K).

We further measured glycolytic function in microglia via extracellular acidification rate, while GW0742 treatment did not change basal glycolysis (Fig. 4A–E), it increased maximal glycolytic capacity (Fig. 4F, G), attributed to an enhanced glycolytic reserve (Fig. 4F). This suggests that GW0742 bolsters microglial capacity to respond to increased energy demands.

Microglia rely on three primary energy substrates for survival: glucose, fatty acids, and glutamine (41). To investigate how GW0742 affects substrate usage, we examined the expression of key genes regulating the uptake and utilization of these substrates in control and GW0742-treated microglial cells, both with or without LPS stimulation (Fig. 4H). We found that GW0742+LPS group showed significantly reduced expression of glucose transporters (*Glut1* and *Glut4*) compared with the DMSO+LPS group (Fig. 4H). Consistent with this, a 2-NBDG uptake assay confirmed that GW0742 reduces glucose uptake in microglia (Supplemental Fig. S2A, B).

In contrast, glutaminase (*Gls*), the rate-limiting enzyme that converts glutamine to glutamate, was further upregulated in the GW0742+LPS group relative to DMSO+LPS controls (Fig. 4H). Meanwhile, LPS reduced the expression of several lipolysis-related genes, including *Cd36*, a fatty acid translocase required for fatty acid import; *Cpt1a*, the rate-limiting enzyme for long-chain fatty acid β-oxidation and a known PPARδ target; and *Lpl*, a triglyceride hydrolase important for receptor-mediated lipoprotein uptake. Because reduced *Lpl* expression is associated with impaired microglial immune responses (8), these findings indicate suppressed lipid uptake in LPS-treated microglia (Fig. 4H). Notably, GW0742 reversed these LPS-induced metabolic changes by enhancing the expression of *Cd36* and *Cpt1a* (Fig. 4H). To validate the functional consequences of this shift toward lipolysis and anti-inflammatory response, we performed BODIPY and TNF-α staining (Fig. 4I). The GW0742+LPS group displayed reduced lipid accumulation and decreased TNF-α levels compared with DMSO+LPS controls (Fig. 4I).

### Synthesis and characterization of NPs-GW0742 for microglial targeting in vitro and in vivo

**Fig. 5:**
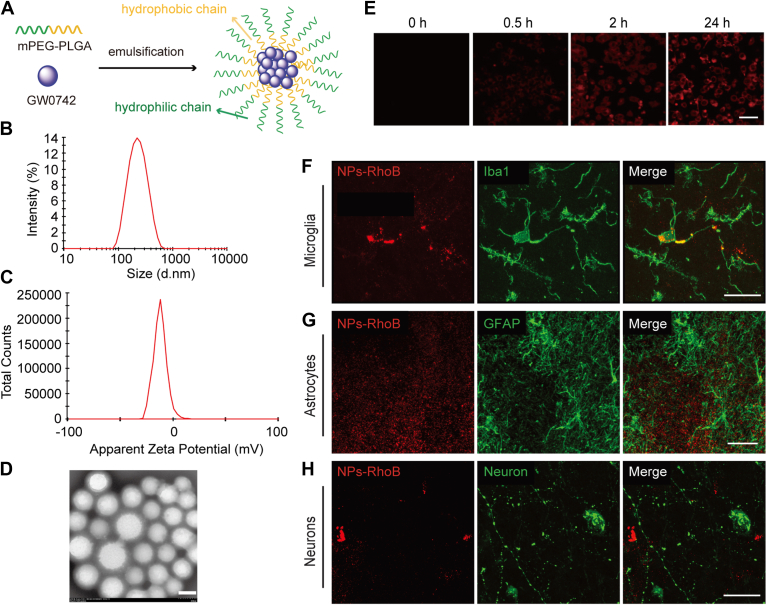
Synthesis and characterization of NPs-GW0742 uptake *in vitro* and *in vivo*. A: Schematic diagram of the synthesis of NPs-GW0742 nanomaterials. B–D: The particle size distribution (B), surface charge (C) and morphology (D) of NPs-GW0742. Scale bar in D, 200 nm. E: NPs-RhoB (red) accumulation in cultured microglial cells after incubation for 0 h, 0.5 h, 2 h or 24 h. Scale bar in E, 50 μm. F: Colocalization of NPs-RhoB (red) and ionized calcium-binding adaptor molecule 1 immunoreactivity (Iba1-ir, green) in microglial cells. G: No NPs-RhoB (red) was detected in astrocytes expressing the glial fibrillary acidic protein-ir (GFAP-ir, green). H: No NPs-RhoB (red) was detected in neurons expressing orexin (Green). n = 3. Scale bar: 20 μm in F, G, 30 μm in H.

The mediobasal hypothalamus (MBH) plays a critical role in regulating systemic energy homeostasis (42). Microglial activation in this region is commonly associated with hypercaloric diet-induced obesity (43). However, small molecule drugs like GW0742 typically diffuse non-specifically throughout the brain after injection, limiting their effectiveness and increasing the risk of off-target effects. To address this, we developed a nanoparticle (NP)-based delivery system designed for selective uptake by microglia in the MBH, enabling targeted and localized drug action. These GW0742-encapsulated nanoparticles (NPs-GW0742) were synthesized using a widely used and biodegradable polymer mPEG-PLGA (Fig. 5A). The resulting NPs-GW0742 exhibited an average diameter of 197.8 nm with a polydispersity index (PDI) of 0.185, indicating a uniform size distribution (Fig. 5B). The surface charge of the NPs-GW0742 was at −12.1 mV (Fig. 5C). The morphology and structural integrity of these NPs were confirmed via transmission electron microscopy, which revealed spherical and uniformly sized particles (Fig. 5D). To assess the cellular uptake of these NPs, we conducted a time-course study in microglial cultures using NPs encapsulated with the fluorescent dye rhodamine B (NPs-RhoB). Over a 24-h period, the RhoB fluorescence intensity per cell increased steadily, demonstrating efficient and sustained NP uptake by microglial cells (Fig. 5E). To verify targeted *in vivo* uptake, we injected NPs-RhoB into the MBH of rat brains and observed the distribution via immunofluorescence staining after 4 h. The results showed that NPs-RhoB selectively accumulated in microglia, while no significant RhoB signal was detected in astrocytes or neurons (Fig. 5F–H).

### Hypothalamic NPs-GW0742 infusion improves insulin sensitivity in HFD-fed rats

**Fig. 6:**
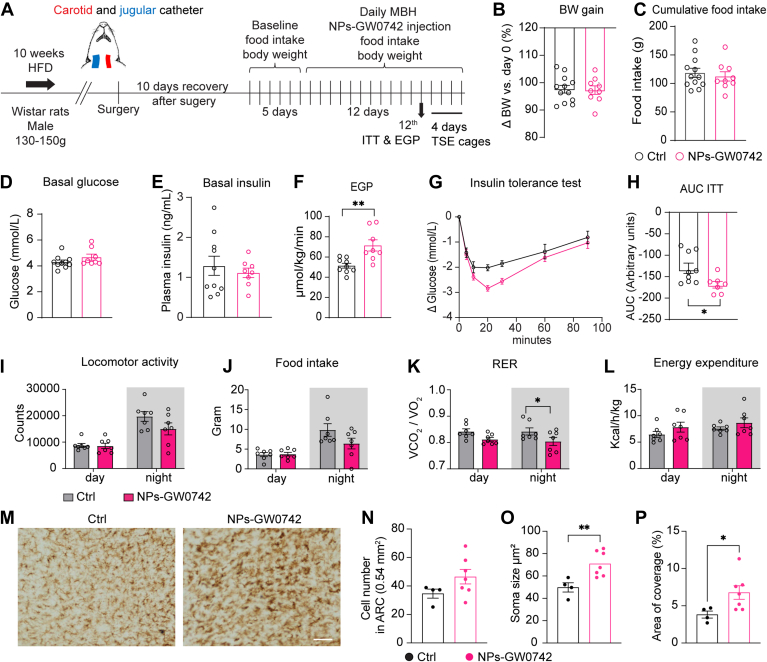
Effects of NPs-GW0742 on microglial activity and insulin sensitivity in rats on a high-fat diet. A: Schematic overview of the *in vivo* experimental design. B, C: Body weight gain and cumulative food intake measured on day 12 following ctrls and NPs-GW0742 infusion into the MBH. D, E: Basal blood glucose and plasma insulin levels on day 12 following infusion. F: Endogenous glucose production (EGP) measured on day 12 following infusion. G, H: Blood glucose dynamics during the insulin tolerance test. I–L: Effects of NPs-GW0742 or Ctrls on diurnal locomotor activity (I), food intake (J), respiratory exchange ratio (RER) (K), and energy expenditure (L) in male Wistar rats. M–P: Morphological changes in microglia, assessed by Iba1 immunoreactivity, following NPs-GW0742 treatment. Scale bar: 50 μm. Data are presented as means ± SEM, with statistical significance indicated as: ∗, *P* < 0.05; ∗∗, *P* < 0.01.

To assess the impact of NPs-GW0742 on food intake, body weight, and insulin sensitivity, we administered NPs-GW0742 daily into the MBH of HFD-fed rats for 12 consecutive days, with blank NPs and NPs monomers serving as controls (Fig. 6A). After the infusion period, no significant differences were observed in body weight or food intake between the NPs-GW0742 and control groups (Fig. 6B, C). Basal blood glucose and insulin levels were also unchanged (Fig. 6D, E), and plasma triglyceride and cholesterol concentrations did not differ between groups (Supplemental Fig. S3A, B). Interestingly, rats treated with NPs-GW0742 exhibited a significantly increased endogenous glucose production and improved insulin sensitivity during an insulin tolerance test (Fig. 6F–H). When animals were housed in the calorimetric cages, NPs-GW0742 did not cause any difference in locomotor activity, food intake, nor energy expenditure, but it did change the respiratory exchange ratio (Fig. 6I–L and Supplemental Fig. S4A–D). We then measured microglial morphological changes in the MBH in response to NPs-GW0742, we found increased microglial activity, characterized by larger soma size and greater microglial cell coverage in the arcuate nucleus of the MBH. Importantly, there was no difference in the total number of microglial cells between the groups (Fig. 6M–P), indicating that the observed morphological changes reflect activation of existing microglia rather than proliferation. This suggests a distinct response compared to previous studies, where an increase in microglial cell number was observed following HFD (3, 4). Together, these results suggest that targeted activation of PPARδ signaling in microglia enhances their functional activity, thereby improving insulin metabolic regulation in diet-induced obesity.

## Discussion

This study underscores the key role of microglial PPARδ activation as an innovative strategy for ameliorating metabolic dysfunction in diet-induced obesity. By employing TPP and *in vitro* functional assays, we demonstrated that the PPARδ agonist GW0742 enhances microglial phagocytic activity, modulates immune responses, and shifts microglial energy metabolism. Furthermore, targeted delivery of GW0742 to microglia in the MBH improved insulin sensitivity in HFD-fed rats, highlighting the therapeutic potential of microglial PPARδ activation in addressing obesity-related metabolic dysfunctions.

Microglia are essential for maintaining neuronal health through phagocytosis and immune surveillance, functions that are compromised in diet-induced obesity (2). Our study builds on previous evidence that impaired lipid metabolism in microglia, such as in lipoprotein lipase deficiency, exacerbates glucose dysregulation and hypothalamic neuron dysfunction in obese mice (8). By activating PPARδ, we observed a significant enhancement in microglial phagocytic activity, which is crucial for clearing cellular debris and maintaining neuronal homeostasis. Our findings are consistent with the established role of PPARδ in promoting fatty acid oxidation and lipid metabolism, processes that are essential for sustaining microglial energy homeostasis and functional integrity in the context of metabolic stress (44).

Microglial immune function is tightly linked to their metabolic state, a concept known as immunometabolism (5). Our results indicate that GW0742 treatment shifts microglial energy metabolism by reducing reliance on oxidative phosphorylation and enhancing glycolytic capacity. This metabolic reprogramming is consistent with the increased phagocytic and immune activity observed in GW0742-treated microglia, as glycolysis provides a rapid energy source necessary for these functions (45). Additionally, the observed reduction in glucose uptake, along with more flexible substrate utilization via metabolism gene expression and lipid droplet staining, suggests a shift from glucose to fatty acids metabolism, potentially optimizing energy use for immune responses (46).

To achieve precise activation of PPARδ in microglia, we developed a nanoparticle-based delivery system (NPs-GW0742) designed to selectively target microglia in the MBH, leveraging their innate phagocytic capacity (23). NPs-GW0742 exhibited efficient cellular uptake and preferential accumulation in microglia, with minimal off-target effects on astrocytes or neurons. Notably, NPs-GW0742 administration improved insulin sensitivity in HFD-fed rats. These results could be attributed to the critical interaction between microglia and pro-opiomelanocortin (POMC) neurons, which play a central role in glucose metabolism and overall energy homeostasis (47). Previous studies have shown that microglia support POMC neurons by maintaining metabolic balance, modulating nutrient availability, and releasing immune signals that influence neuronal activity (47, 48, 49). Moreover, microglia contribute to the structural and functional integrity of POMC neurons by modulating synaptic pruning and plasticity, both of which are important for effective signal transmission and neuropeptide release in response to metabolic cues (49, 50). However, under HFD conditions, chronic microglial pro-inflammatory states disrupt POMC function and impair insulin signaling, leading to metabolic imbalances (48). Our findings indicate that activating PPARδ with GW0742 induces an anti-inflammatory state in microglia, reducing cytokine release and inflammation. This shift potentially restores POMC neuron function and improves glucose metabolism.

While our findings demonstrate that GW0742 altered microglial immunometabolism and microglial-targeted delivery of NPs-GW0742 improves insulin sensitivity in HFD-fed rats, several limitations should be acknowledged. First, we acknowledge that using 2-NBDG as a surrogate for glucose uptake is a methodological limitation, as recent studies question its specificity for GLUT-mediated transport and its ability to accurately reflect physiological glucose flux (51, 52). Because 2-NBDG differs from native glucose in transporter affinity, uptake mechanisms, and intracellular handling, the quantitative interpretation of our measurements should be viewed with caution (51, 52). Future studies using complementary methods, such as radiolabelled glucose uptake or metabolic flux assays, will be good for validating these observations. Second, we also acknowledge that the effects of GW0742 on microglial outcomes, such as phagocytosis and cytokine regulation, may not be exclusively mediated through metabolic pathways. PPARδ is known to regulate a broad range of processes in the periphery, including inflammatory signaling and autophagy (53, 54). It cannot be fully excluded that the differential effects of the different PPARδ agonists on specific outcomes may be due to PPARδ-independent mechanisms, considering the relatively high concentration of PPARδ agonists used. Besides, the animal treatment duration was limited to 12 days, which may have been insufficient to observe changes in body weight or food intake. It is possible that longer-term administration is required to elicit more substantial effects on energy balance. Moreover, while microglial activation improved insulin sensitivity, the complex neuroendocrine networks governing body weight regulation may not have been sufficiently influenced by microglial-specific PPARδ activation alone. This highlights the need to investigate combinatorial approaches that also modulate peripheral or neuronal pathways. Lastly, given that lifestyle factors such as diet and exercise are known to influence microglial function and metabolism (55, 56), future investigations should examine whether combining GW0742 treatment with lifestyle interventions enhances therapeutic outcomes. Such combined strategies may offer a more effective and sustainable approach to treating obesity and related metabolic disorders.

## Data Availability

Data are available from the corresponding authors on reasonable request.

## Supplemental Data

This article contains supplemental data.

## Conflict of Interest

The authors declare that they have no conflicts of interest with the contents of this article.
