# Supplementary material for: Targeted PPARδ activation reprograms microglial immunometabolism and improves insulin sensitivity in HFD-fed rats
Source: J Lipid Res. 2026 Jan 8;67(2):100978. doi: 10.1016/j.jlr.2026.100978 (PMC12874120; doi:10.1016/j.jlr.2026.100978)
Supplement: Supplemental Information [file mmc1.docx]

**SUPPLEMENTAL INFORMATION**

**Title: Targeted PPARδ activation reprograms microglial immunometabolism and improves insulin sensitivity in HFD-fed rats**

Han Jiao^1,2,3,4#^, Fernando Cázarez-Márquez^1,2,3,4,5#^, Valentina Sophia Rumanova^1,2,3,4^, Yalin Wang^6^, Andries Kalsbeek^1,2,3,4^, Gertjan Kramer^7^, Shanshan Guo^6,8^*, Chun-Xia Yi^1,2,3,4^*

1. Department of Endocrinology and Metabolism, Amsterdam University Medical Center, location AMC, University of Amsterdam, Amsterdam, The Netherlands.
2. Amsterdam Gastroenterology Endocrinology and Metabolism, Amsterdam, The Netherlands.
3. Department of Clinical Chemistry, Laboratory of Endocrinology, Amsterdam University Medical Centers, location AMC, Amsterdam, The Netherlands.
4. Netherlands Institute for Neuroscience, Amsterdam, The Netherlands.
5. Department of Arctic and Marine Biology, UiT The Arctic University of Norway, Tromsø, Norway.
6. Key Laboratory of Quantitative Synthetic Biology, Shenzhen Institute of Synthetic Biology, Shenzhen Institutes of Advanced Technology, Chinese Academy of Sciences, Shenzhen, China.
7. Department of Mass Spectrometry of Biomolecules, Swammerdam Institute for Life Sciences, University of Amsterdam, Amsterdam, the Netherlands.
8. Faculty of Synthetic Biology, Shenzhen University of Advanced Technology, Shenzhen, China.

^#^ These authors contributed equally to this study.

* Corresponding Authors

**CONTENTS:**

**Supplemental Fig. S1-S4**.

**Supplemental Table. S1**

**Supplemental Fig. S1:** Cell viability of microglial cells was almost not affected by the 24 h PPARδ agonists. A-B: The cell viability of GNF-0242 (A), GNF-8501 (B) and GW0742 (C) treated microglial cells. Data are presented as means ± SD.

**Supplemental Fig. S2:** The 2-NBDG glucose uptake assay

A-B: The representative figure and quantification of GW0742 treated group compared to vehicle control group. Scale bar: 30 μm. Data are presented as means ± SEM and statistical significance was determined using Unpaired t test. ***, p<0.001

**Supplemental Fig. S3:** NPs-GW0742 did not alter the plasma triglycerides and cholesterol concentration.

A-B: The plasma triglycerides and cholesterol concentration. Data are presented as means ± SEM.


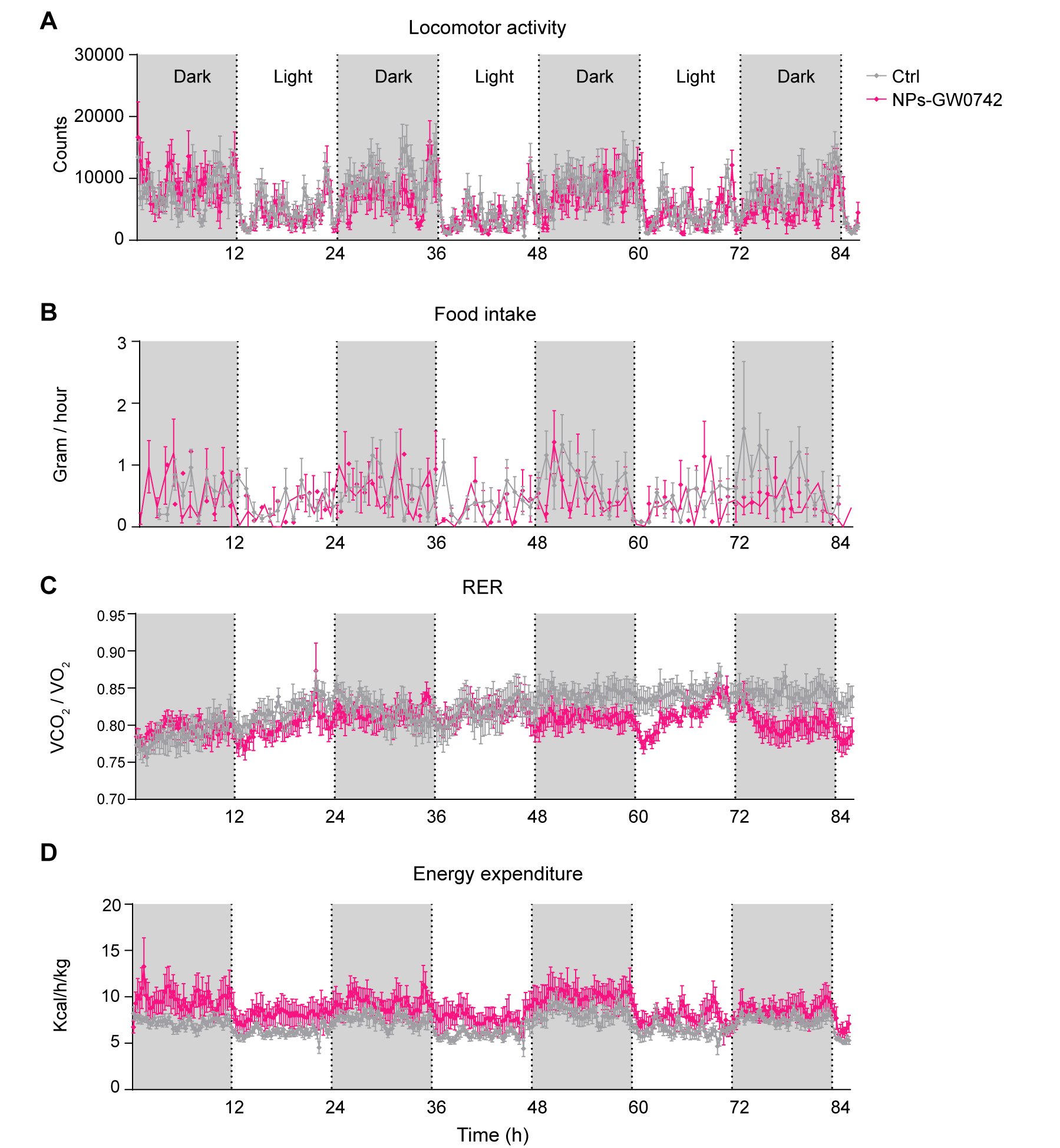


**Supplemental Fig. S4**. Indirect calorimetry data in rats treated with the NPs-GW0742.

*A-D:* Effects of NPs-GW0742 or controls (blank NPs and NPs monomers) on (A) locomotor activity, (B) food intake, (C) respiratory exchange ratio (RER), and (D) energy expenditure in male Wistar rats on 3.5 consecutive days. Data are presented as means ± SEM.

| **Primer** | **Forward 5’ to 3’** | **Reverse** |
| --- | --- | --- |
| *Tnfα* | TCTCATCAGTTCTATGGCCC | GGGAGTAGACAAGGTACAAC |
| *Il6* | GTTCTCTGGGAAATCGTGGA | TGTACTCCAGGTAGCTATGG |
| *Il10* | ATGCAGGACTTTAAGGGTTACTTG | TAGACACCTTGGTCTTGGAGCTTA |
| *iNos/Nos2* | ACATCGACCCGTCCACAGTAT | CAGAGGGGTAGGCTTGTCTC |
| *Gls* | TACGACTCCAGAACAGCCCT | TTATTCCACCTGTCCTTGGGG |
| *Cd36* | ACAGTTTTGGATCTTTGACGTG | CCTTGGCTAAATAACGAACTCTG |
| *Glut4* | TCCCTTCAGTTTGGCTATAACATTG | ACGTTGCATTGTAGCTCTGTTCA |
| *Glut1* | ACGTCCATTCTCCGTTTCAC | TCCCACGGCCAACATAAG |
| *Cpt1a* | ACAATGGGACATTCCAGGAG | AAAGACTGGCGCTGCTCA |
| *Lpl* | CAAAACAACCAGGCCTTCGA | AGCAATTCCCCGATGTCCA |
| *Gapdh* | CTCCCACTCTTCCACCTTCG | CCTCTCTTGCTCAGTGTCCT |
| *Hprt* | GCAGTACAGCCCCAAAATGG | AACAAAGTCTGGCCTGTATCCAA |
| *Rpl27* | TCGTGAAGAACATTGACGAT | CGCTTCAAAGCTGGGTCCCT |

**Supplemental Table. S1.** RT-PCR primer sequences.
